# Effect of crude *Ganoderma applanatum* polysaccharides as a renoprotective agent against carbon tetrachloride-induced early kidney fibrosis in mice

**DOI:** 10.14202/vetworld.2022.1022-1030

**Published:** 2022-04-23

**Authors:** Raden Joko Kuncoroningrat Susilo, Dwi Winarni, Suhailah Hayaza, Ruey-An Doong, Sri Puji Astuti Wahyuningsih, Win Darmanto

**Affiliations:** 1 Department of Biology, Faculty of Science and Technology, Universitas Airlangga, Surabaya 60115, Indonesia; 2 Institute of Analytical and Environmental Sciences, National Tsing Hua University, Sec. 2 Kuang Fu Road, Hsinchu 30013, Taiwan; 3 Institute of Science Technology and Health, Jl. Kemuning 57A, Jombang, Indonesia

**Keywords:** anti-inflammatory, carbon tetrachloride, fibrosis, *Ganoderma applanatum*, kidney

## Abstract

**Background and Aim:**

Interstitial fibrosis is the final stage of chronic kidney injury, which begins with an inflammatory process. Crude *Ganoderma applanatum* polysaccharides are known to have anti-inflammatory properties. The potential role of crude *G. applanatum* polysaccharides in renal fibrosis through pro-inflammatory cytokines needs further investigation. This study aimed to determine the renoprotective effect of crude *G. applanatum* polysaccharide extract in mice with carbon tetrachloride (CCL_4_)-induced early kidney fibrosis.

**Materials and Methods:**

This study was conducted for 4 weeks using 24 male BALB/c mice selected for their metabolic stability. The mice were randomly divided into six groups, including control (CG), model (MG), silymarin group and crude *G. applanatum* polysaccharide extract groups comprising doses of 25, 50, and 100 mg/kg body weight. After sacrificing the mice, whole blood was analyzed for urea and creatine levels, and kidney tissue was prepared to assess tumor necrosis factor-α (TNF-α), interleukin-6 (IL-6), hyaluronic acid (HA), and laminin levels, both using enzyme-linked immunosorbent assay. Kidney histology was determined using hematoxylin and eosin staining, while the extracellular matrix (ECM) components were stained using Masson’s trichome staining. The α-smooth muscle actin (α-SMA) concentration was determined using immunohistochemistry. These parameters were measured to determine the effectiveness of the crude *G. applanatum* polysaccharide extract in preventing interstitial fibrosis.

**Results:**

Administration of crude *G. applanatum* polysaccharides effectively prevented increases in kidney weight and physiological enzymes, pro-inflammatory cytokines, and ECM production compared with those in the MG, as evidenced by the low levels of urea, creatinine, TNF-α, IL-6, HA, and laminin. Histopathological results also showed that crude *G. applanatum* polysaccharides prevented the occurrence of inflammatory infiltration, desquamated nuclei, cytoplasm debris, rupture at the brush border, dilatation of the glomeruli space and lumen of the proximal tubule, and necrotic cells compared with the MG. Masson’s trichrome staining revealed lower collagen levels in the interstitial tubules of kidney tissue than those in the MG. Immunohistochemical analysis revealed low α-SMA expression in the crude *G. applanatum* polysaccharides treatment groups than that in the MG.

**Conclusion:**

The crude polysaccharide extract of *G. applanatum* has a protective effect that prevents the progression of kidney fibrosis in mice.

## Introduction

Chronic kidney disease (CKD) is among the most common causes of death worldwide, with approximately 850,000 deaths annually [1-3]. This disease is often accompanied by kidney fibrosis, which is involved in the end stage of CKD, with abundant extracellular matrix (ECM) deposition, including collagen, hyaluronic acid (HA), and laminin in the tubulointerstitium. Nephron injury and an increase in myofibroblasts are common in patients with renal fibrosis, leading to a decline in kidney function [4,5]. Research on fibrosis increases our knowledge of physiology, histopathology, and biochemical processes occurring during CKD, making it easier to determine means to prevent it. Several factors affect the pathological process of fibrosis, especially the inflammation pathway, including pro-inflammatory cytokine elevation [6,7]. Studies on renal fibrosis have experimentally confirmed that the increased number of inflammatory cells in the tubulointerstitium is related to the severity of renal fibrosis [8]. This demonstrates the importance of the inflammatory pathway in renal fibrosis development [9]. In response to injury, pro-inflammatory cytokines such as tumor necrosis factor-α (TNF-α) and interleukin-6 (IL-6) are increased to trigger the transdifferentiation of fibroblasts and epithelial and endothelial cells into myofibroblasts to produce ECM [10]. Especially in kidney tissue, epithelial cells are transformed into myofibroblasts during the epithelial-mesenchymal transition (EMT) process to begin interstitial fibrosis with an abundance of ECM [11,12]. Therefore, preventive treatment is important to suppress renal fibrosis. Fibrosis can be induced by carbon tetrachloride (CCl_4_), which is often used to construct organ toxicity models based on free radical activity [13]. This alteration destroys the permeability of the mitochondria, plasma membrane, and endoplasmic reticulum in the cell and causes DNA fragmentation. Furthermore, CCl_4_ has been reported to enhance cholesterol, free fatty acid, and triglyceride levels [14].

Natural products such as fungal and plant polysaccharides are often used for preventive therapy, including that of renal fibrosis [15]. Polysaccharides are often obtained from plants and fungi because of their fewer side effects and considerable bioactivity [16]. *Ganoderma applanatum* is a medicinal mushroom with several biological activities such as immunomodulatory, anti-tumor, antioxidant, hypoglycemic, hypolipidemic, hepatoprotective, and antiviral activities [17,18]. Hence, crude *G. applanatum* polysaccharide is suitable for preventing renal fibrosis. However, new types of natural products and their mechanisms of renoprotection against fibrosis remain to be investigated. Moreover, early-stage intervention increases the likelihood of successful renal fibrosis prevention [8].

Therefore, this study aimed to determine the renoprotective effects of crude *G. applanatum* polysaccharide extract on early kidney fibrosis in mice.

## Materials and Methods

### Ethical approval

This research was approved by the Laboratory Animal Care Guidance of the Ethical Committee of the Faculty of Veterinary Medicine, Universitas Airlangga (2.KE.168.10.2018).

### Study period and location

This study was conducted in March 2021 at the Department of Biology, Faculty of Science and Technology, Universitas Airlangga, Indonesia.

### Preparation of crude extract

The basidiocarp *G. applanatum* was obtained from Tulungagung City Forest Park, East Java, Indonesia. This mushroom was identified by Dr. Ni’matuzahroh of the Department of Biology, Faculty of Science and Technology, Universitas Airlangga, Indonesia. The extraction procedure was performed according to Susilo *et al*. [19]. Briefly, 500 g of pounded *G. applanatum* was heated in 10 L of distilled water at 75°C for 6 h with occasional stirring, and the extract was filtered using Whatman filter paper (Merck, Darmstadt, Germany). The filtrate was then centrifuged at 2000× *g* for 10 min, and the supernatant was precipitated twice with absolute ethanol at a ratio of 1:4 (w/v). Thereafter, the extract was centrifuged again as described above, and the residue was dissolved in distilled water. Finally, the extract was lyophilized and stored at –20°C until further use.

### Animals

Twenty-four healthy male BALB/c mice (selected for their metabolic stability) aged 2-3 months old, weighing 30-40 g were provided by the Faculty of Pharmacy, Universitas Airlangga, Surabaya, Indonesia, for use in the experiments. All 24 mice were acclimatized for 7 days and exposed to a 12 h day/night lighting cycle, with temperature maintained at 22±2°C and humidity in the range of 60-70%. Feed and water were provided *ad libitum*.

### Experimental design

The mice (n=24) were randomly divided into six experimental groups of four mice each [20]. The study was conducted for 4 weeks. The control group (CG) was treated with distilled water only, while the model group (MG) was induced using CCl_4_ only (Merck) at 2 mL/kg. The low crude *G. applanatum* polysaccharide group was treated with crude *G. applanatum* polysaccharide extract at a dose of 25 mg/kg body weight (BW) and CCl_4_ (2 mL/kg BW); the moderate crude *G. applanatum* polysaccharide group was treated with crude *G. applanatum* polysaccharide extract at a dose of 50 mg/kg BW and CCl_4_ (2 mL/kg BW); and the high crude *G. applanatum* polysaccharide group was treated with crude *G. applanatum* polysaccharide extract at a dose of 100 mg/kg BW and CCl_4_ (2 mL/kg BW). The silymarin group (SG) was treated with a dose of 100 mg/kg silymarin extract and CCl_4_ (2 mL/kg BW). CCl_4_ solution was dissolved in olive oil at a ratio of 1:3 and administered twice a week for 4 weeks intraperitoneally. In contrast, crude *G. applanatum* polysaccharide and silymarin extracts were diluted in distilled water and orally administered every day for 4 weeks. At 24 h after 4 weeks, the mice were anesthetized by intramuscular injection with ketamine (15 mg/kg BW) and xylazine (2 mg/kg BW) and sacrificed to collect whole blood and kidney tissue.

### Enzyme-linked immunosorbent assay (ELISA) test

After whole blood was collected intracardially, it was centrifuged at 1000× *g* at 8°C for 20 min to produce supernatant for urea and creatinine evaluation. Meanwhile, the acquired kidney tissue was homogenized using a sonication ultrasound homogenizer (Eppendorf, Hamburg, Germany) and then centrifuged at 1000× *g* at 8°C for 20 min. Thereafter, the tissue supernatant was used to determine TNF-α, IL-6, laminin, and HA. All measurements were performed according to the manufacturer’s instructions for the ELISA kit (Nanjing Jiancheng Biotechnology Co., Ltd., Nanjing, China).

### Hematoxylin and eosin (HE) staining

The kidney tissue samples were immersed in 10% neutral buffered formalin for 48 h to preserve the samples. Thereafter, dehydration was performed by dipping in alcohol graded from 70% to absolute, followed by embedding in xylol paraffin (1:1) for 30 min, thrice in paraffin for 60 min each time, and cutting the sample to 5 mm thickness. Afterward, the slides were cleared with xylol for 10 min, rehydrated in reverse alcohol (absolute to 70% each for 5 min), and stained with H&E (Sigma-Aldrich, St. Louis, MO, USA) for histological analysis. Finally, slides were mounted using Entellan (Merck). All kidney injury parameters, including necrosis and hydropic cells, were observed under an inverted microscope (Olympus IX51; Olympus Corporation, Tokyo, Japan).

### Immunohistochemical analysis

The immunohistochemistry kit was purchased from Elabscience (Houston, Texas, USA). After rehydration, the slides were immersed in citrate base (pH 6.0) for the antigen masking process, followed by incubation in blocking solution (3% H_2_O_2_) for 10 min for endogenous peroxidase blocking. Subsequently, the slides were washed with phosphate-buffered saline (PBS; Merck) for 10 min and incubated again with normal blocking serum. Next, the slides were incubated with goat anti-mouse α-smooth muscle actin (α-SMA) for 30 min and biotinylated donkey anti-goat IgG for 30 min. Afterward, the slides were washed with PBS for 5 min. Finally, 3,3-diaminobenzidine (DAB) and H_2_O_2_ were used to visualize α-SMA. The slides were counterstained with hematoxylin.

### Masson’s trichome staining

Masson’s trichrome staining was performed according to the manufacturer’s instructions (Polysciences, Warrington, USA). Briefly, after xylol cleaning and rehydration with reverse gradient alcohol, the slides were washed with distilled water. The slides were then stained with hematoxylin for 10 min and washed again in distilled water. Next, the sections were immersed in aniline blue solution (Sigma-Aldrich) for 10 min, rinsed in distilled water for 5 min, and washed again in distilled water. Finally, they were mounted using Entellan (Merck).

### Statistical analysis

All data are presented as the mean±SD (n=4) and were analyzed using GraphPad Prism (version 8.0; GraphPad Software, San Diego, CA, USA). The Shapiro–Wilk test was applied to test for normality. Kidney weight, urea, creatinine, TNF-α, IL-6, HA, and laminin levels were assessed using one-way ANOVA followed by Tukey multiple comparison tests. Statistical significance was set at p<0.05.

## Results

### Effect of crude *G. applanatum* polysaccharide on kidney biochemical and inflammatory cytokine parameters

**Figure-1:**
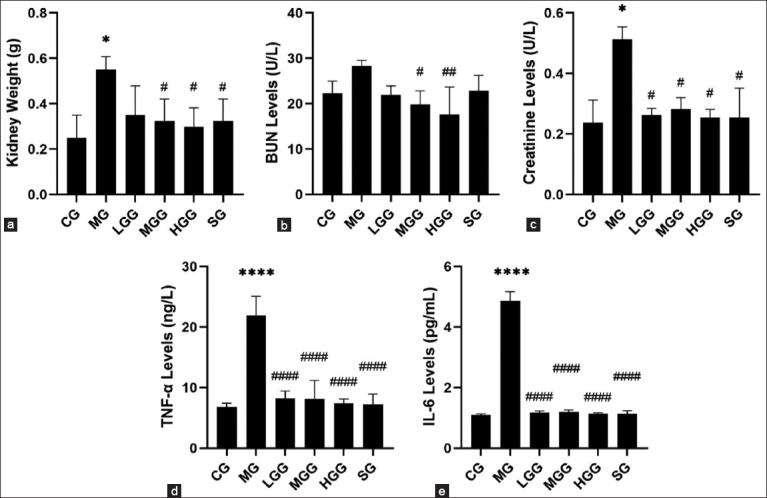
Effects of crude *Ganoderma applanatum* polysaccharide extract on (a) kidney weight (b) urea levels, (c) creatinine levels, (d) TNF-α levels, and (e) IL-6 levels. Data are presented as mean±SD (n=4 in each group). The column bar graph shows the results of the Tukey test analysis. Different superscript letters indicate significant differences. *p<0.05 compared with the control group (CG). ^#^p<0.05 compared with the model group (MG). ^##^p<0.01 compared with the MG. ****p<0.001 compared with the CG. ^####^p<0.001 compared with the MG. CG, control; MG, CCl_4_ only; LGG, CCl_4_+25 mg/kg crude *G. applanatum* polysaccharide extract; MGG, CCl_4_+50 mg/kg crude *G. applanatum* polysaccharide extract; HGG, CCl_4_+100 mg/kg crude *G. applanatum* polysaccharide extract; and silymarin group, CCl_4_+silymarin extract; BUN=Blood urea nitrogen, TNF-α=Tumor necrosis factor-α, IL-6=Interleukin-6.

As shown in Figure-1, urea levels in the MG were not significantly different (p=0.1888) from those in the CG. However, several parameters, such as kidney weight (p=0.0286), creatinine levels (p=0.0286), TNF-α levels (p<0.0001), and IL-6 levels (p<0.0001), in the MG were significantly higher than those in the CG. Administration of silymarin and moderate and high crude *G. applanatum* polysaccharides significantly prevented (p=0.0286, 0.0286, and 0.0286, respectively) kidney weight gain, but low crude *G. applanatum* polysaccharide did not significantly (p=0.0857) prevent kidney weight gain. Furthermore, administering silymarin and low, medium, and high crude *G. applanatum* polysaccharides prevented an increase in creatinine levels (p=0.0286, 0.0286, 0.0286, and 0.0286, respectively), TNF-α levels (all p<0.0001), and IL-6 levels (all p<0.0001) compared with the MG. However, silymarin treatment did not significantly (p=0.2660) prevent an increase in the aforementioned parameters. These results indicated that crude *G. applanatum* polysaccharides effectively played a protective role against kidney fibrosis. Comparison of the effects of silymarin and crude *G. applanatum* polysaccharides on kidney weight and creatinine, TNF-α, and IL-6 levels did not show significant differences. However, urea levels indicated that crude *G. applanatum* polysaccharide doses of 50 and 100 mg/kg were more effective than silymarin treatment. Meanwhile, among the doses of *G. applanatum* administered to mice, the 100 mg/kg dose showed the highest effectiveness compared with the other *G. applanatum* doses, as evidenced by the lowest inhibition of increases in kidney weight and urea, creatinine, TNF-α, and IL-6 levels.

### Effects of crude *G. applanatum* polysaccharide on kidney histopathology

**Figure-2:**
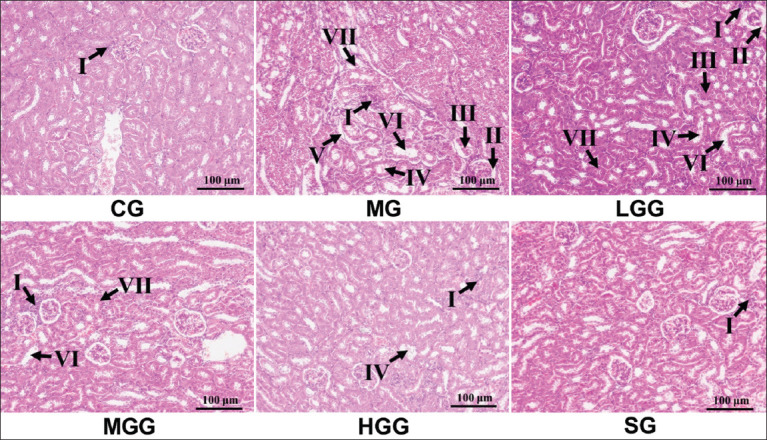
Effect of crude *Ganoderma applanatum* polysaccharide extract on histological appearance through HE staining at 200×. Kidney tissue injury is indicated by the presence of (I) inflammatory infiltration, (II) desquamated nuclei, (III) cytoplasm debris, (IV) rupture at the brush border, (V) dilatation of the glomerular space, (VI) dilatation at the lumen of the proximal tubule, (VII) and necrotic cells. CG, control; MG, CCl_4_ only; LGG, CCl_4_+25 mg/kg crude *G. applanatum* polysaccharide extract; MGG, CCl_4_+50 mg/kg crude *G. applanatum* polysaccharide extract; HGG, CCl_4_+100 mg/kg crude *G. applanatum* polysaccharide extract; and silymarin group, CCl_4_+silymarin extract.

**Figure-3:**
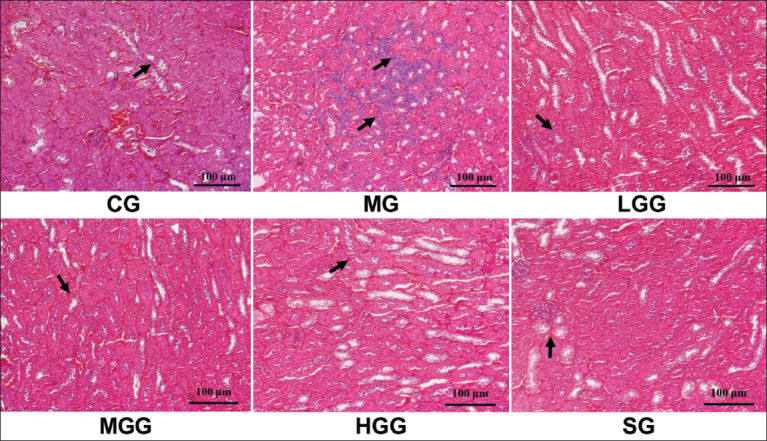
Effect of crude *Ganoderma applanatum* polysaccharide extract on histologic appearance through Masson’s trichome staining at 200×. The black arrows show blue-stained collagen areas. CG, control; MG, CCl_4_ only; LGG; CCl_4_+25 mg/kg crude *G. applanatum* polysaccharide extract; MGG, CCl_4_+50 mg/kg crude *G. applanatum* polysaccharide extract; HGG, CCl_4_+100 mg/kg crude *G. applanatum* polysaccharide extract; and silymarin group, CCl_4_+silymarin extract.

This study also explored the histopathological aspect of kidney tissue shape as a basic method to detect kidney injury. As shown in Figure-2, some injuries were observed after CCl_4_ induction. In the MG, we found inflammatory cells in the interstitial renal tubules, as well as desquamated nuclei, cytoplasmic debris in the lumen of renal tubules, and some ruptures at the brush border of the renal tubules. Moreover, there was a large dilatation in the glomerular space and the lumen of the renal tubule, and considerable necrosis was discovered in the renal tubule cells. Silymarin and crude *G. applanatum* polysaccharide treatments prevented the enhancement of inflammatory cells, as evidenced by their low levels. However, silymarin and crude *G. applanatum* polysaccharides did not eliminate dilatation of renal tubules and glomeruli space, with several injuries being found, albeit some of the injuries being reduced. The same was true for cytoplasmic debris and necrotic cells. Regarding desquamated nuclei and rupture at the brush border, administration of silymarin and crude *G. applanatum* polysaccharides prevented the emergence of these injuries as they were absent at the time of observation. H&E staining clarified that silymarin and crude *G. applanatum* polysaccharide administration prevented kidney injury but not as perfect as CG Meanwhile, the comparison between the silymarin and crude *G. applanatum* polysaccharide treatments showed that crude *G. applanatum* polysaccharides reduced kidney damage parameters but was not as effective as silymarin which only showed inflammatory infiltration. Increasing crude *G. applanatum* polysaccharide dose displayed better results, with less kidney damage. Furthermore, as shown in Figure-3, Masson’s trichrome staining was used to determine the collagen content in kidney tissue. After CCl_4_ induction, the MG showed a strongly blue-stained area in the interstitial renal tubules, indicating that collagen was expressed. Thus, the MG exhibited interstitial fibrosis in the renal tubules. Conversely, silymarin and crude *G. applanatum* polysaccharide treatments showed fewer blue-stained areas, indicating that both treatments prevented collagen production from myofibroblasts in the renal tubule. Treatment with crude *G. applanatum* polysaccharides and silymarin displayed similar Masson’s trichome staining results, indicating that both treatments had the same potential against kidney fibrosis. Similarly, all crude *G. applanatum* polysaccharide treatments did not show clear differences in Masson’s trichome staining, implying that they all had a similar effect. A large amount of collagen found following Masson’s trichome staining indicated that ECM was the primary constituent of interstitial fibrosis in the renal tubule. The impact of giving silymarin and *G. applanatum* protected kidney cells from interstitial fibrosis.

### Effects of crude *G. applanatum* polysaccharide on α-SMA expression and HA and laminin levels

**Figure-4:**
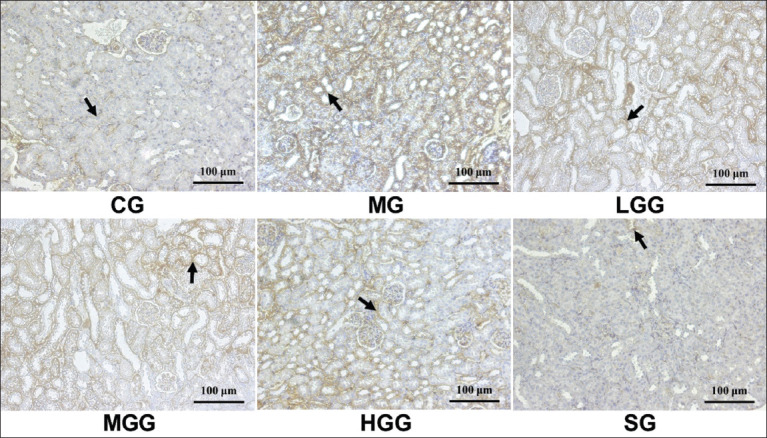
Effect of crude *Ganoderma applanatum* polysaccharide extract on the α-SMA expression from immunohistochemical analysis at 200×. The black arrows indicate brown-stained α-SMA expression areas. CG, control; MG, CCl_4_ only; LGG, CCl_4_+25 mg/kg crude *G. applanatum* polysaccharide extract; MGG, CCl_4_+50 mg/kg crude *G. applanatum* polysaccharide extract; HGG, CCl_4_+100 mg/kg crude *G. applanatum* polysaccharide extract; and silymarin group, CCl_4_+silymarin extract; α-SMA=α-Smooth muscle actin.

**Figure-5:**
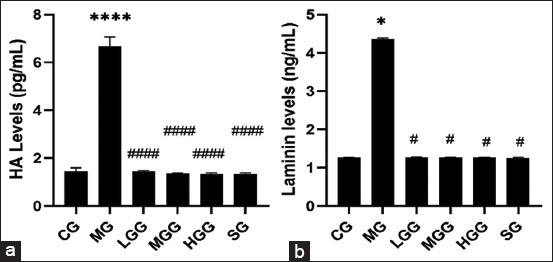
Effects of crude *Ganoderma applanatum* polysaccharide extract on (a) HA levels and (b) laminin levels. Data are presented as mean±SD (n=4 in each group). The column bar graph presents the results of the Tukey test analysis. Different superscripts indicate significant differences. *p<0.05 compared with the control group (CG). ^#^p<0.05 compared with the model group (MG). ****p<0.001 compared with the CG. ^####^p<0.001 compared with the MG. CG, control; MG, CCl_4_ only; LGG, CCl_4_+25 mg/kg crude *G. applanatum* polysaccharide extract; MGG, CCl_4_+50 mg/kg crude *G. applanatum* polysaccharide extract; HGG, CCl_4_+100 mg/kg crude *G. applanatum* polysaccharide extract; and silymarin group, CCl_4_+silymarin extract; HA=Hyaluronic acid.

Reversal of myofibroblasts differentiation has become the most important therapeutic strategy for fibrosis treatment after it was considered most responsible for producing ECM. Hence, studies on EMT are urgently required to develop strategies for ameliorating fibrosis. As shown in Figure-4, the MG showed a higher expression of α-SMA than the other groups. This suggests that CCl_4_ could induce myofibroblasts to produce ECM, whereas α-SMA acts as a myofibroblast marker. Moreover, in the crude *G. applanatum* polysaccharide treatment groups, α-SMA expression was observed in some renal tubules; however, it decreased relative to that in the MG, indicating that treatment with crude *G. applanatum* polysaccharides could prevent α-SMA expression. The α-SMA expression following treatment with 100 mg/kg of crude *G. applanatum* polysaccharides was slightly higher than that of the 25 and 50 mg/kg crude *G. applanatum* polysaccharide treatments. The SG exhibited superior α-SMA expression prevention than the crude *G. applanatum polysaccharide* treatment groups. This could be seen from the exiguous α-SMA expression, and it also showed that α-SMA expression almost resembled that of the CG. These results indicate that the administration of silymarin and crude *G. applanatum* polysaccharides could inhibit the action of myofibroblasts. Long ECM is a major component of interstitial fibrosis in the renal tubules, and identifying its constituent components, such as HA and laminin, can also indicate the severity of fibrosis. As results shown in Figure-5, induction of CC_l4_ only in MG significantly increased HA (p<0.0001) and laminin levels (p=0.0286) compared with the CG. Preventive action was observed in the silymarin, low, moderate, and high crude *G. applanatum* polysaccharide treatment groups with significantly inhibited HA (all p<0.0001) and laminin levels (p=0.0286, 0.0286, 0.0286, and 0.0286, respectively). Both silymarin and crude *G. applanatum* polysaccharide treatments had the same effect on HA and laminin levels; thus, there was no significant difference between the two groups. Furthermore, there were no significant differences in HA and laminin levels among the crude *G. applanatum* polysaccharide treatments. Hence, the protective effects of silymarin and crude *G. applanatum* polysaccharides impacted HA and laminin levels.

## Discussion

Kidney fibrosis is the final stage of CKD, in which kidney tissue restoration occurs by producing a large amount of ECM in interstitial tubules [21]. ECM is produced by myofibroblasts originating from the tubular epithelium of the kidney. This process, which directs the tubular epithelium in an interstitial direction, is known as EMT. As a result of EMT, several ECMs can accumulate in interstitial tubules section [22-24]. The occurrence of CKD is due to induction by CCl_4_, which causes toxicity in renal tubular cells. CCL_4_ is a toxic substance that is harmful to various organs, such as the liver, kidney, heart, and intestine [25,26].

This study aimed to determine the protective effect of crude *G. applanatum* polysaccharide extract against early kidney fibrosis induced by CCl_4_. Biochemical analysis showed that CCl_4_ induction significantly increased kidney weight and physiological parameters, such as urea and creatinine levels, indicating that the impact of CCl_4_ metabolism in the body triggers glomerular cell and kidney tubule toxicity [27,28]. An increase in these parameters resulted in abnormalities in the glomerular cells and renal tubules. Treatment with silymarin and crude *G. applanatum* polysaccharides prevented an increase in kidney weight and creatinine levels, whereas crude *G. applanatum* polysaccharides could only prevent an increase in urea levels. Injury to glomerular cells and kidney tubules leads to the production of pro-inflammatory cytokines by inflammatory cells, initiating the regeneration of injured cells [29,30].

Toxic substances such as CCl_4_ prolong glomerular cell and kidney tubule injuries, causing the production of pro-inflammatory cytokines such as TNF-α and IL-6 to occur continuously [31,32]. Excessive exposure to TNF-α and IL-6 disrupts the normal regeneration process and induces the EMT process, which activates myofibroblasts to produce ECM to fill space caused by injuries in glomerular cells and proximal tubule cells [33,34]. The continued increase in TNF-α and IL-6 levels was inhibited in the silymarin and crude *G. applanatum* polysaccharide groups. Both compounds showed an anti-inflammatory role, thereby indirectly preventing kidney injury. We used histopathological observation with HE staining as the basic method to determine the degree of injury to the glomerular cells and kidney tubules. Accordingly, CCl_4_ induction showed inflammatory cell infiltration into the kidney tissue, which indicates injury to the cells composing the tissue in that area. In addition, CCl_4_ induction also causes desquamated nuclei, cytoplasm debris, rupture at the brush border, and dilatation of the proximal tubular lumen due to CCl_4_ toxicity in damaged cell parts.

Glomeruli also experience adverse effects from CCl_4_ toxicity in the form of dilatation of the outer membrane, allowing disruption of nutrient filtration that the body still needs. Several kidney tubule cells also experience necrosis, which affects kidney tubule physiology. Some of these effects cause acute inflammation initiated by effector cells [35]. The anti-inflammatory effects of silymarin and crude *G. applanatum* polysaccharides play a role in preventing glomerular and renal tubular injury, which, in this case, is determined by a few parameters of kidney injury, such as dilatation of both the glomerulus and renal tubules, necrosis, cytoplasmic debris, and inflammatory infiltration into the interstitial tubules. Masson’s trichome staining showed the amount of collagen deposited in the interstitial tubules after exposure to CCl_4_ – blue staining confirmed collagen deposition in the kidney tissue. Collagen deposition in the interstitial tubules results from myofibroblast activation, which acts as a major producer of ECM. Meanwhile, treatment with silymarin and crude *G. applanatum* polysaccharides protected kidney tissue by limiting collagen production, thus blocking the occurrence of interstitial fibrosis.

Myofibroblast activation can be seen from α-SMA expression as the primary marker of myofibroblast activation [36]. Thus, CCl_4_ induction also affects the EMT process which activates myofibroblasts to produce ECM which is proved by increasing of HA and laminin levels [37,38]. HA and laminin are glycoproteins that are constituents of the ECM in kidney fibrosis [39,40]. The presence of HA and laminin also confirms that CCl_4_ induction in the interstitial portion of the renal tubules contains several ECMs that are characteristic of interstitial fibrosis. The anti-inflammatory effects of silymarin and crude *G. applanatum* polysaccharides were also observed with the inhibition of increasing α-SMA expression. This is related to the low levels of pro-inflammatory cytokines measured previously; thus, the stimulatory effect is reduced by accelerating myofibroblast action in producing ECM [41,42]. In addition, the levels of HA and laminin, as part of the ECM, were low in the silymarin and crude *G. applanatum* polysaccharide treatment groups, confirming that the low inflammation levels signified little potential for interstitial fibrosis. Thus, the crude *G. applanatum* polysaccharides tested in this study were protective against CCl_4_-induced interstitial fibrosis in kidney tissue.

## Conclusion

This study determined the protective effect of crude *G. applanatum* polysaccharides in preventing kidney fibrosis in mice by inhibiting significant increases in kidney physiological parameters, pro-inflammatory cytokines, and ECM components compared with MG. We demonstrated that the greatest renoprotective effect was obtained by treatment with 100 mg/kg BW of crude *G. applanatum* polysaccharide extract. Therefore, crude *G. applanatum* polysaccharides can be categorized as alternative materials for preventing interstitial fibrosis. The results of this study are useful as a basis for clinical trials in kidney fibrosis research using natural plant resources. However, this study has some limitations such as no mRNA evaluation and NF-kB pathway. Therefore, in the future, it is necessary to further investigate the relationship between genes and fibrosis parameters.

## Authors’ Contributions

RJKS, SH, and WD: Planned, designed, and evaluated the study. RJKS and SH: Did study work, collected organs, analyzed data, made crude *G*. *applanatum* polysaccharide extracts, prepared material and drafted the manuscript. SAH, DW, SPAW, RD, and DW: Supervised the study and revised the manuscript. All authors made a substantial contribution to the research. All authors read and approved the final manuscript.
